# TEM1 expression in cancer-associated fibroblasts is correlated with a poor prognosis in patients with gastric cancer

**DOI:** 10.1002/cam4.515

**Published:** 2015-09-04

**Authors:** Satoshi Fujii, Ayano Fujihara, Kei Natori, Anna Abe, Yasutoshi Kuboki, Youichi Higuchi, Masaki Aizawa, Takeshi Kuwata, Takahiro Kinoshita, Wataru Yasui, Atsushi Ochiai

**Affiliations:** 1 Pathology Division, Research Center for Innovative Oncology National Cancer Center at Kashiwa, National Cancer Center Hospital East6-5-1 Kashiwanoha, Kashiwa, Chiba, 277-8577, Japan; 2 Department of Digestive Surgery, Niigata Cancer Center Hospital2-15-3 Kawagishicho, Chuou-ku, Niigata, 951-8566, Japan; 3 Department of Surgical Oncology, National Cancer Center Hospital East6-5-1 Kashiwanoha, Kashiwa, Chiba, 277-8577, Japan; 4 Department of Molecular Pathology, Hiroshima University Institute of Biomedical and Health SciencesMinami-ku, Hiroshima, 734-8551, Japan

**Keywords:** Cancer stromal cell, cancer-associated fibroblast, gastric cancer, immunohistochemistry, TEM1

## Abstract

The cancer stroma, including cancer-associated fibroblasts (CAFs), is known to contribute to cancer cell progression and metastasis, suggesting that functional proteins expressed specifically in CAFs might be candidate molecular targets for cancer treatment. The purpose of the present study was to explore the possibility of using TEM1 (tumor endothelial marker 1), which is known to be expressed in several types of mesenchymal cells, as a molecular target by examining the impact of TEM1 expression on clinicopathological factors in gastric cancer patients. A total of 945 consecutive patients with gastric cancer who underwent surgery at the National Cancer Center Hospital East between January 2003 and July 2007 were examined using a tissue microarray approach. TEM1 expression in CAFs or vessel-associated cells was determined using immunohistochemical staining. Three items (CAF-TEM1-positivity, CAF-TEM1-intensity, and vessel-TEM1-intensity) were then examined to determine the correlations between the TEM1 expression status and the recurrence-free survival (RFS), overall survival (OS), cancer-related survival (COS), and other clinicopathological factors. Significant correlations between CAF-TEM1-positivity or CAF-TEM1-intensity and RFS, OS, or COS were observed (*P *<* *0.001, Kaplan–Meier curves); however, no significant correlation between vessel-TEM1-intensity and RFS, OS, or COS was observed. A univariate analysis showed that CAF-TEM1-positivity and CAF-TEM1-intensity were each correlated with a scirrhous subtype, tumor depth, nodal status, distant metastasis, serosal invasion, lymphatic or venous vessel infiltrations, and pTMN stage. This study suggests that the inhibition of TEM1 expression specifically in the CAFs of gastric carcinoma might represent a new strategy for the treatment of gastric cancer.

## Introduction

Cancer tissue consists of cancer cells and stromal cells, such as fibroblasts, endothelial cells, and inflammatory cells including macrophages. Cancer-associated fibroblasts (CAFs) are well known to contribute to cancer cell progression and metastasis 1. That is, CAFs are a positive regulator of cancer aggressiveness, suggesting that CAFs might be a useful target cell type and that functional proteins expressed specifically in CAFs might also be candidate targets for molecular-targeted therapy. Therefore, proteins that are specifically overexpressed in CAFs and that are associated with a poor prognosis must be identified to enable the development of innovative molecular targeting drugs.

Tumor endothelial marker 1 (TEM1), which is also called endosialin or CD248, is located at 11q13.2 and is a transmembrane protein for which thrombomodulin and C1qRp are homologs 2. Mouse Tem1 is expressed throughout embryonic and adult development in several types of mesenchymal cells that are closely related to blood vessels 3. Human TEM1 is also known to be highly expressed in human CAFs and bone marrow-derived mesenchymal stem cells 4. On the other hand, tumor-related neovascular smooth muscle cells are known to express TEM1 in malignant tumors in humans; moreover, a role of TEM1 in tumor development via an interaction between cancer cells and CAFs has been reported 5. A metastasis-related protein, Mac2 BP/90K, on tumor cells has been reported to be a specific ligand of TEM1 on stromal fibroblasts, suggesting that a bidirectional interaction between tumor cells, such as neuroblastoma and stromal fibroblasts, might be an important mechanism for tumor development 5. Human TEM1 is 77.5% identical with mouse Tem1, which is expressed in mouse fetal fibroblasts and endothelial cells; however, its expression decreases in neonates, and no expression is observed in adult mice. Interestingly, a report on mouse Tem1 showed that the size of malignant tumors transplanted into abdominal sites in Tem1-knockout mice was smaller than that in wild-type mice, and this difference in size was correlated with poor metastasis and a longer survival 6. This previous report also showed that the expression of Tem1 in stromal fibroblasts controls tumor aggressiveness, which has significant implications for tumor invasiveness. On the other hand, human endosialin/TEM1 overexpression has been observed in many cancer cells of various tissue origins, including the colon, breast, pancreas, and lung 7; however, no report has focused on the expression of TEM1 in both cancer and stromal cells in human cancer tissue, nor have the clinical implications, such as the effect on overall survival (OS) and recurrence-free survival (RFS), especially in patients with gastric cancer, been examined. In the current study, we examined the effects of TEM1 expression on clinicopathological factors, including OS and RFS, in patients with gastric cancer to explore the possibility that TEM1 might be a useful molecular target for therapy.

## Materials and Methods

### Patients

A total of 945 consecutive and available patients who underwent surgical operations between January 2003 and July 2007 were selected from the case files of the National Cancer Center Hospital East and enrolled in this study. Cases with neoadjuvant therapy were excluded in advance of the selection. The clinicopathological records were reviewed and evaluated retrospectively after the institutional review board of the National Cancer Center approved all the protocols for the patients’ agreements. The pathological stage was determined according to the International Union Against Cancer UICC-TNM classification (seventh edition). Adjuvant chemotherapy was administered to 60 (6.34%) patients.

### Pathological examination

The resected specimens were fixed in 10% formalin at room temperature. The tumor-containing sections were processed routinely and embedded in paraffin. Serial sections of the paraffin-embedded blocks for each tumor were cut and stained with hematoxylin and eosin (H&E); the sections were then examined to confirm the pathological diagnosis. Elastica staining was used to check for blood vessel infiltration as part of a routine analysis.

### Tissue microarray

The representative tumor areas were selected and marked on H&E-stained slides, and the corresponding paraffin blocks were prepared for the construction of a tissue microarray (TMA). Duplicate cylindrical cores containing the representative tumor area with a diameter of 2.0 mm were punched out from the corresponding block for each case using a manual tissue arrayer (Azumaya Ika Kikai, Tokyo, Japan) and assembled in a TMA format. A total of 23 specimens and a dummy tissue were assembled in a TMA format. A section cut from each TMA block was stained with H&E and was then examined to confirm the histology. Serial 4-*μ*m thick sections were used for immunohistochemical staining.

### Immunohistochemical staining

Immunohistochemical staining was performed using antiendosialin antibody (mouse monoclonal, Clone B1/35, Cat. No. MAB2626, Lot No. 2049281, 1:200 dilution [5 *μ*g/mL]; Millipore, Billeria, MA). Microwaving was selected as the optimal method for antigen retrieval after trials of several methods were performed, and the EnVision system (Dako, Tokyo, Japan) was used for detection followed by counterstaining with hematoxylin. The validation of this antibody was performed using several methods as follows: qRT-PCR, western blot analysis, and immunofluorescence and immunohistochemical staining using sections cut from the paraffin embedded blocks of MKN7 and SS7 cells after formalin fixation, which were used to examine the expression of *TEM1* mRNA and TEM1 protein.

### Mouse animal model with peritoneal dissemination of gastric cancer cell lines

Female BALB/c (5-week-old) nude mice were purchased from CLEA Japan, Inc. (Tokyo, Japan). The animals were maintained under conventional housing conditions. All the animal experiments were conducted in accordance with the principles and procedures outlined in the National Institutes of Health’s Guide for the Care and Use of Laboratory Animals. The protocols for the animal experiments were approved by the Animal Experimentation Committee of the National Cancer Center. The human gastric cancer cell line, HSC-44PE (a kind gift from Dr. Kazuyoshi Yanagihara who established this cell line) was cultured in RPMI 1640 medium supplemented with 10% heat-inactivated FBS (Fetal Bovine Serum), 100 units of penicillin, and 100 *μ*g/mL streptomycin at 37°C under 5% CO_2_. The construction of a luciferase-expressing plasmid and luciferase gene-transfected HSC-44PE (HSC-44PE/LUC) cells were performed as follows. Cloned luciferase cDNA was inserted into the multicloning site of a CSII-CMV-MCS-IRES2-Bsd vector. The CSII-CMV-MCS-IRES2-Bsd encoding LUC and a control vector, CSII-CMV-MCS-IRES2-Bsd, were transfected into HSC-44PE cells using a lentivirus system. The HSC-44PE/LUC cells with spontaneous-metastatic potential (1 × 10^7^ cells suspended in 0.2 mL of serum-free RPMI 1640) were inoculated into 6-week-old female BALB/c nude mice intraperitoneally. Three weeks later, the peritoneal dissemination of the HSC-44PE/LUC cells was visualized using Living Image 3.2 (Caliper Life Science, Hopkinton, Mass, NA, New England); the animals were then sacrificed by cervical dislocation. The total RNA was extracted from the metastatic tumor in the peritoneum or the peritoneum without a metastatic tumor. Total RNA from the tumors was isolated using TRIzol Reagent (Invitrogen, Carlshad, CA) and reverse transcribed to cDNA using ExScript RT Reagent (TaKaRa, Kusatsu, Shiga, Japan) after DNase treatment. Quantitative real-time RT-PCR was performed using specific primers for mouse *Tem1* and *Gapdh* and a Smart Cycler (Cepheid, Sunnyvale, CA). Mouse *Gapdh* expression was used to normalize the results for variance. Real-time fluorescence monitoring of the PCR products was performed using SYBR Green I fluorescent dye (TaKaRa). The levels of expression of specific genes were reported as ratios to the expression level of mouse *Gapdh* in the same master reaction. The PCR primer pairs (5′ to 3′) used for each gene were as follows: mouse *Tem1*, AGCAGATGGGCACAGTTGTG and AAGGCGACAGTGGCAGCTA; and mouse *Gapdh*, AAATGGTGAAGGTCGGTGTG and TGAAGGGGTCGTTGATGG.

### Cell lines

Six cell lines including SS7 (primary fibroblast from serosa of gastric cancer), VAF (primary vascular adventitial fibroblast from lung cancer) 8, HeLa (cervical cancer cell line; cervical adenocarcinoma), MKN7 (gastric cancer cell line; well differentiated tubular adenocarcinoma), TE4 (esophageal cancer cell line; well differentiated squamous cell carcinoma), and DLD1 (colorectal cancer cell line; adenocarcinoma) were cultured and used for the experiments as described below. The HeLa and DLD1 cell lines were purchased from American Type Culture Collection (ATCC) in 2005 and 2011, respectively. The MKN7 cell line was purchased from Immuno-Biological Laboratories Co., Ltd. (Gunma, Japan) in 2012. The TE4 cell line was a gift from Dr. Katsuya Tsuchihara, who purchased it from the RIKEN BioResource Center (Tsukuba, Ibaraki, Japan) in 2010. These cell lines had been stocked in liquid nitrogen. In the current study, we resuscitated these cell lines from the stocks for the experiment and used them in less than 2 months after resuscitation. The all cell lines had been routinely tested and were negative for mycoplasma. Human primary fibroblasts, SS7, were obtained from surgically resected gastric tissues located around the pylorus at a point 5 cm distant from a stomach tumor. To separate the subserosal layer of gastric tissue, the first muscular layer was detached from the tissues and the mucosal layer was scrubbed away using sterile tweezers and scissors. The peritoneum of the stomach was then peeled away to obtain the subserosal tissue. The residual tissue lump was washed with PBS (Phosphate Buffered Saline), then incubated with trypsin, and the subserosal fibroblasts (SS7) were finally obtained 9. The SS7 cells were grown and maintained in MF-medium (Toyobo, Osaka, Japan). The VAF cells were obtained, grown, and maintained in our laboratory as described previously 8. Moreover, we established noncancer-associated fibroblasts (NCAFs) (StSMFs2; stomach submucosal fibroblasts 2) and CAFs (StCAFs2; stomach cancer-associated fibroblasts 2) from gastric cancer tissue of the same patient. These cell lines were grown and maintained as described earlier.

### RNA isolation and quantitative real-time RT-PCR for human cancer or mesenchymal cells

As described earlier, the total RNA from each of the various cell lines including SS7, VAF, HeLa, TE4, DLD1, StSMFs2, and StCAFs2, was isolated and qRT-PCR was performed to examine the expressions of human *TEM1* or *GAPDH* in these cell lines. The PCR primer pairs (5′ to 3′) used for each gene were as follows: human *TEM1*, TTGCACTGGGCATCGTGTA and TTGCTCCCAGCATGGATGAC; and human *GAPDH*, GCACCGTCAAGGCTGAGAAC and ATGGTGGTGAAGACGCCAGT.

### Western blot analysis

The cancer or mesenchymal cells were lysed with whole-cell lysis buffer, and the protein was extracted as described previously 10. The protein concentration of each sample was determined using a BCA™ Protein Assay Kit (Pierce, Rockford, IL). Equal amounts of protein (20 *μ*g) from each cell line were subjected to a western blot analysis. The probing antibodies were antiendosialin antibody (1:2500 dilution), which was the same antibody used for the immunohistochemistry, and *β*-actin antibody (1:100 dilution; goat polyclonal, C-11, sc-1615; Santa Cruz Biotechnology, Inc., Dallas, TX).

### Cell block

MKN7 and SS7 cells were fixed in 10% formalin and embedded in paraffin. Sections with a thickness of 4 *μ*m were used for immunohistochemical staining according to the same procedure as that used for immunohistochemical staining for the TMA.

### Immunofluorescence

Cells grown on coverslips were fixed with 10% formalin and processed for immunofluorescence as described previously 11. The probing antibody was antiendosialin antibody (1:200 dilution; 5 *μ*g/mL), which was the same antibody as that used for the immunohistochemistry. The secondary antibody was an Alexa Fluor 488 F(ab’)2 fragment of goat antimouse IgG (H+L) (Molecular Probes, Life Technologies Corporation, Carlsbad, CA). The cells were examined using a fluorescence microscope (BZ-9000 BIOREVO; Keyence, Osaka, Japan).

### Evaluation of the TEM1 expression level in CAFs using immunohistochemical staining

The pericytes in a noncancerous area were used as a positive internal control for the immunohistochemical staining of TEM1 for each section, since the pericytes reportedly express TEM1 12,13. Three items, CAF-TEM1-positivity, CAF-TEM1-intensity, and vessel-TEM1-intensity, were evaluated using the TMA. CAF-TEM1-positivity was determined based on the percentage of CAFs with TEM1 expression among all the CAFs in the cancer tissue. For CAF-TEM1-intensity, the TEM1-expression level of the CAFs was classified into four categories: grade 0, no TEM1-expression in CAFs in the cancer tissue; grade 1, the TEM1 expression-intensity of the CAFs was weaker than that of the internal control cells; grade 2, the TEM1 expression-intensity of the CAFs was almost the same as that of the internal control cells; and grade 3, the TEM1 expression-intensity of the CAFs was stronger than that of the internal control cells. Moreover, the vessel-TEM1-intensity was judged as described above, with the highest TEM1-expression level of the vessel-associated cells including pericyte, vascular adventitial fibroblast, and vascular smooth muscle cell in the cancer tissue classified into four categories: grade 0, no TEM1 expression in the vessel-associated cells described above in the cancer tissue; grade 1, the TEM1 expression-intensity of the vessel-associated cells was weaker than that of the internal control cells; grade 2, the TEM1 expression-intensity of the vessel-associated cells was almost the same as that of the internal control cells; and grade 3, the TEM1 expression-intensity of the vessel-associated cells was stronger than that of the internal control cells.

### Statistical analysis

The relationships between the estimated TEM1 expression level, as evaluated in terms of CAF-TEM1-positivity, CAF-TEM1-intensity, and vessel-TEM1-intensity, and the clinicopathological factors were examined. The chi-square test was used to compare the covariates among the patient groups classified according to the TEM1 expression level. The OS curves and the RFS curves were drawn using the Kaplan–Meier method. Differences were determined using a log-rank test if the TEM1 expression levels were classified into two categories. The univariate and multivariate analyses using the Cox proportional hazard model were performed to identify factors influencing the RFS, OS, and cancer-related survival (COS) in patients who underwent curative surgical resection. The statistical analyses were performed using Dr. SPSS, ver.20 (IBM Japan, Tokyo, Japan).

## Results

### Patient characteristics

Of a total of 1006 cases, 945 cases that had not received neoadjuvant chemotherapy and whose sections were available for immunohistochemical analysis were enrolled in this study. The clinicopathological factors are summarized in Table1. The median age of the patients at the time of the diagnosis of gastric carcinoma was 64 years (range, 18–92 years). Four hundred and fifty (47.6%) patients were classified as having tubular adenocarcinoma, including well- or moderately differentiated subtypes, and 323 (34.2%) were classified as having poorly differentiated adenocarcinoma. Twenty-two, 130, and 20 patients were diagnosed as having papillary adenocarcinoma, signet ring cell carcinoma, and mucinous carcinoma, respectively. Sixty (6.3%) patients received adjuvant therapy. All 945 patients were followed for survival, and the follow-up period was measured from the date of surgery until the date of the last confirmation of the patient’s status. The median follow-up period was 1826 days. Overall, 147 patients were diagnosed as having local recurrence and metastasis during the follow-up period. One hundred and twenty-six patients died of their disease. Recurrence was defined as initial tumor recurrence or metastasis, which was considered to be evidence of tumor relapse, and all deaths including gastric cancer deaths and deaths from other diseases that the patients had (OS) or cancer-related deaths (COS) were considered for the purposes of this study.

**Table 1:** Clinicopathological factors of 945 patients with gastric cancer

Gender	
Male/female	630/315
Age, years	
Median/range	64/18–92
Histological subtype, *n* (%)	
Papillary adenocarcinoma	22 (2.3)
Tubular adenocarcinoma well differentiated type	154 (16.2)
Tubular adenocarcinoma moderately differentiated type	296 (31.3)
Poorly differentiated adenocarcinoma solid type	75 (7.9)
Poorly differentiated adenocarcinoma nonsolid type	248 (26.2)
Signet ring cell carcinoma	130 (13.8)
Mucinous	20 (2.1)
Macroscopic tumor type, *n* (%)	
Type 0	500 (53.0)
Type 1	25 (2.6)
Type 2	116 (12.3)
Type 3	239 (25.3)
Type 4	50 (5.3)
Type 5	15 (1.6)
Pathological tumor depth, *n* (%)	
T1	485 (51.3)
T2	116 (12.3)
T3	209 (22.1)
T4	135 (14.3)
Pathological nodal status, *n* (%)	
N0	585 (62.0)
N1–3	359 (38.0)
Nx^1^	1 (0.1)
Distant metastasis	
Positive/negative	63/882
Serosal invasion	
Positive/negative	134/811
Lymphatic vessel infiltration	
Present/absent	404/541
Venous vessel infiltration	
Present/absent	437/508
Vessel infiltration^2^	
Present/absent	531/414
Pathological TNM stage, *n* (%)	
Stage I	519 (55.0)
Stage II	203 (21.5)
Stage III	160 (16.9)
Stage IV	63 (6.7)
Resection margin, *n* (%)	
R0	904 (95.2)
R1	45 (4.8)
Neoadjuvant chemotherapy, *n* (%)	
Present/absent	0/945
Adjuvant chemotherapy, *n* (%)	
Present/absent/no data	60/883/2

1 Nx: unknown for nodal status.

2 Vessel infiltration including venous vessel infiltration and/or lymphatic vessel infiltration.

### CAFs at peritoneal metastatic sites express Tem1

**Figure 1:**
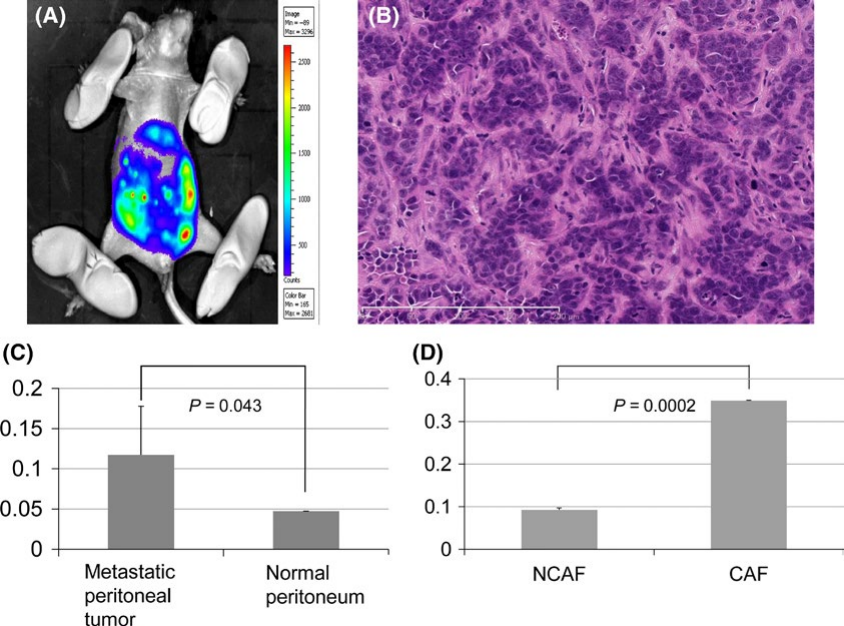
Mouse animal model with peritoneal dissemination of gastric cancer cells. (A) Peritoneal dissemination of HSC-44PE gastric cancer cells detected using fluorescence. (B) Histology of peritoneal metastatic tumor (H&E staining). (C) *Tem1* mRNA expression level of peritoneal metastatic tumor as determined using qRT-PCR. A *P*-value less than 0.05 was considered statistically significant. (D) *TEM1* mRNA expression levels of NCAF and CAF cells as determined using qRT-PCR. PCR reaction was performed twice to validate the reproducibility of this experiment. A *P*-value less than 0.05 was considered statistically significant. H&E, hematoxylin and eosin; *Tem1*, tumor endothelial marker 1; NCAF, noncancer-associated fibroblast.

The peritoneal dissemination of gastric cancer cells was confirmed using fluorescence in a mouse animal model (Fig.1A). The histology of the peritoneal disseminations consisted of both gastric cancer cells and stromal cells, mainly including mouse fibroblastic cells called CAFs and also including pericytes and endothelial cells (Fig.1B). The qRT-PCR revealed that the CAFs strongly expressed mouse *Tem1* mRNA, compared with normal fibroblasts in the peritoneum (Fig.1C), because mouse *Tem1* primers were designed for detecting mouse *Tem1* mRNA specifically. These results showed that the activated fibroblasts, which were called CAFs, expressed *Tem1* mRNA. To examine the specific expression of TEM1 mRNA in CAFs, we established the CAF and NCAF cell lines from gastric cancer tissue of the one patient and performed the comparison of *TEM1* mRNA expression between paired CAF and NCAF cell lines from the same patient shown in Figure1D. The results showed that *TEM1* mRNA expression level of CAFs was significantly higher than that of NCAFs (*P *=* *0.0002).

### Validation of the antiendosialin (TEM1) antibody using qRT-PCR, immunofluorescence staining, and immunohistochemical staining

**Figure 2:**
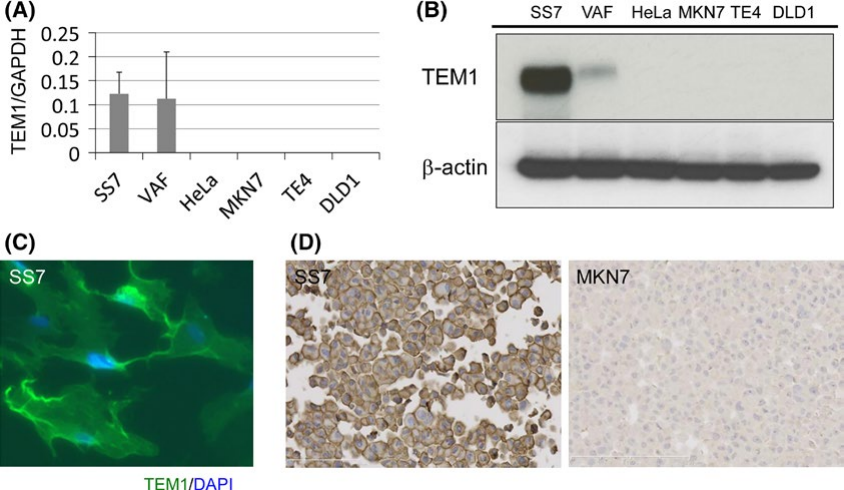
Validation of tumor endothelial marker 1 (TEM1) antibody for immunohistochemical staining. (A) *TEM1* mRNA expression in cell lines including SS7, VAF, HeLa, MKN7, TE4, and DLD1, as detected using qRT-PCR. (B) TEM1 protein expression in cell lines including SS7, VAF, HeLa, MKN7, TE4, and DLD1, as detected using western blot analyses. (C) TEM-1 protein in SS7 cells as detected using immunofluorescence staining. (D) TEM1 protein in SS7 cells as detected using immunohistochemical staining, and the absence of TEM1 protein in MKN7 cells.

The qRT-PCR analysis revealed that SS7 and VAF expressed *TEM1* mRNA, while the cancer cells, including HeLa, MKN7, TE4, and DLD1, did not express *TEM1* mRNA (Fig.2A). The western blot analysis also revealed that SS7 and VAF expressed TEM1 protein, while the cancer cells, including HeLa, MKN7, TE4, and DLD1, did not express TEM1 protein (Fig.2B), which was consistent with the qRT-PCR results, as described above. The MKN7 and SS7 cells were fixed with formalin and embedded in paraffin. The thin sections cut from these cell blocks were subjected to immunofluorescence staining and immunohistochemical staining (Fig.2C and D). The immunofluorescence staining detected TEM1 in the cell membrane of the SS7 cells, which was compatible with the localization of TEM1 (Fig.2C). The immunohistochemical staining showed that SS7 expressed TEM1 in the cell membrane, whereas MKN7 (without *TEM1* mRNA and TEM1 protein expressions) did not express TEM1 by immunohistochemical staining (Fig.2D). These results confirmed that the antiendosialin antibody detected TEM1 protein appropriately, thereby validating the use of this antibody.

### TEM1 expression in the CAFs of gastric cancer tissues was observed using immunohistochemical staining

**Figure 3:**
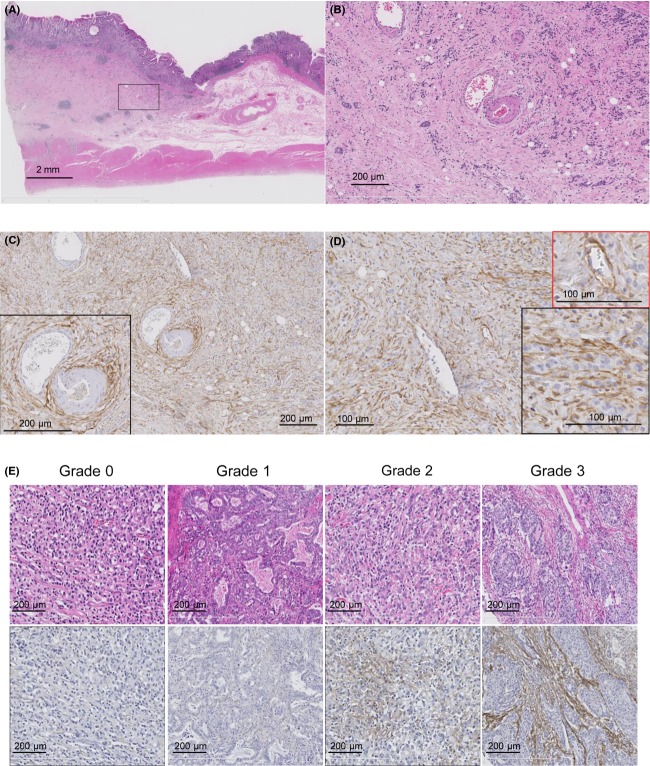
TEM1 expression in human gastric cancer tissue and grading of CAF1-TEM1-intensity in gastric cancer tissue. (A) Scirrhous-type gastric adenocarcinoma showing the thickening of the submucosal layer was accompanied by desmoplasia consisting of CAF proliferation (H&E staining). (B) The magnified photomicrograph at the black frame of (A) (H&E staining). The small clusters of adenocarcinoma cells were observed accompanied by proliferation of CAFs. (C) TEM1-expressing stromal cells were observed in both the lamina propria and the submucosal layer as shown at the same portion as (B). The vessel adventitial fibroblast and vascular smooth muscle cell within the black frame expressed TEM1 protein. (D) The magnified photomicrograph of (C). The pericytes within the red frame expressed TEM1 protein. The CAF within the black frame expressed TEM1 protein, whereas gastric adenocarcinoma cells with plump nucleus within the black frame did not express TEM1. (E) The CAF-TEM1-intensity was classified into four categories, grade 0, grade 1, grade 2, and grade 3, as shown in the representative photomicrographs. The grading systems were described in the Materials and Methods section. TEM1, tumor endothelial marker 1; CAF, cancer-associated fibroblast; H&E, hematoxylin and eosin.

Scirrhous-type gastric carcinoma showed a thickening of the gastric wall, accompanied by desmoplasia (Fig.3A). The CAFs in the lamina propria and submucosal layer were shown to express TEM1 protein using immunohistochemical staining (Fig.3B). The vascular smooth muscle cell, vascular adventitial fibroblast, and pericyte in the submucosal layer also showed TEM1 protein expression (Fig.3B and C). The photomicrograph in Figure3D shows the cytoplasmic expression of TEM1 in the CAFs, however, did not show any expression of TEM1 in the adenocarcinoma cells.

### Grading of the TEM1 expression level using immunohistochemical staining

For the CAF-TEM1-intensity, the TEM1-expression level of CAF was classified into four categories (grade 0, grade 1, grade 2, and grade 3) as described in the Materials and Methods section. Representative photomicrographs are shown in Figure3E.

### Relationships between CAF-TEM1-positivity, CAF-TEM1-intensity, or vessel-TEM1-intensity and clinicopathological factors

The relationships between CAF-TEM1-positivity, CAF-TEM1-intensity, and vessel-TEM1-intesity and various clinicopathological factors are shown in Tables2 and S1, respectively. CAF-TEM1-positivity was more commonly observed in patients with scirrhous-type carcinoma (*P *=* *0.024), a pathological depth of penetration (*P *<* *0.0001), pathological nodal status (*P *<* *0.0001), distant metastasis (*P *=* *0.034), serosal invasion (*P *=* *0.013), lymphatic vessel infiltration (*P *<* *0.0001), venous vessel infiltration (*P *<* *0.0001), vessel infiltration (*P *<* *0.0001), and pTNM stage (*P *<* *0.0001), as shown in Table2. CAF-TEM1-intensity was more commonly observed in patients with scirrhous-type cancer (*P *<* *0.0001), a pathological depth of penetration (*P *<* *0.0001), pathological nodal status (*P *<* *0.0001), distant metastasis (*P *=* *0.013), serosal invasion (*P *<* *0.001), lymphatic vessel infiltration (*P *<* *0.0001), venous vessel infiltration (*P *<* *0.0001), vessel infiltration (*P *<* *0.0001), pTNM stage (*P *<* *0.0001), and resection margin (*P *=* *0.036), as shown in Table2. Vessel-TEM1-intensity was more commonly observed in patients with a pathological depth of penetration (*P *<* *0.0001), pathological nodal status (*P *=* *0.002), lymphatic vessel infiltration (*P *<* *0.001), venous vessel infiltration (*P *<* *0.001), vessel infiltration (*P *<* *0.0001), and pTNM stage (*P *=* *0.011), as shown in Table S1.

**Table 2:** Relationship between CAF-TEM1-positivity, CAF-TEM1-intensity, and clinicopathological factors in 945 patients with gastric cancer

	CAF-TEM1-positivity			CAF-TEM1-intensity		
	<50 (%)*n* (%)	≥50 (%)*n* (%)	*P* value	0–2*n* (%)	3*n* (%)	*P* value
Total number	711	234		623	322	
Age (years)			*P *=* *0.227			*P *=* *0.537
<65	383 (53.9)	115 (49.1)		333 (53.5)	165 (51.2)	
≥65	328 (46.1)	119 (50.9)		290 (46.5)	157 (48.8)	
Gender			*P *=* *0.936			*P *=* *0.041
Male	473 (66.5)	157 (67.1)		401 (64.4)	229 (71.1)	
Female	238 (33.5)	77 (32.9)		222 (35.6)	93 (28.9)	
Histological subtype, *n* (%)			*P *=* *0.292			*P *=* *0.100
Differentiated subtypes	348 (48.9)	124 (53.0)		299 (48.0)	173 (53.7)	
Poorly differentiated/signet ring/mucinous	363 (51.1)	110 (47.0)		324 (52.0)	149 (46.3)	
Diffuse type			*P *=* *0.408			*P *=* *0.131
Diffuse	354 (49.8)	124 (53.0)		304 (63.6)	174 (36.4)	
Undiffuse	357 (50.2)	110 (47.0)		319 (68.3)	148 (31.7)	
Scirrhous stomach cancer			*P *=* *0.024			*P *<* *0.0001
Positive	29 (4.1)	19 (8.1)		18 (2.9)	30 (9.3)	
Negative	682 (95.9)	215 (91.9)		605 (97.1)	292 (90.7)	
Pathological depth of penetration, *n* (%)			*P *<* *0.0001			*P *<* *0.0001
T1	430 (60.5)	55 (23.5)		404 (64.8)	81 (25.2)	
T2	71 (10.0)	45 (19.2)		59 (9.5)	57 (17.7)	
T3	121 (17.0)	88 (37.6)		93 (14.9)	116 (36.0)	
T4	89 (12.5)	46 (19.7)		67 (10.8)	68 (21.1)	
Pathological nodal status, *n* (%)			*P *<* *0.0001			*P *<* *0.0001
N0	479 (67.4)	106 (45.3)		442 (70.9)	143 (44.4)	
N1–3	231 (32.5)	128 (54.7)		181 (29.0)	178 (55.3)	
Nx^1^	1 (0.1)	0 (0.0)		0 (0.0)	1 (0.3)	
Distant metastasis			*P *=* *0.034			*P *=* *0.013
Positive	40 (5.6)	23 (9.8)		32 (5.1)	31 (9.6)	
Negative	671 (94.4)	211 (90.2)		591 (94.9)	291 (90.4)	
Serosal invasion			*P *=* *0.013			*P *<* *0.001
Positive	89 (12.5)	45 (19.2)		68 (10.9)	66 (20.5)	
Negative	622 (87.5)	189 (80.8)		555 (89.1)	256 (79.5)	
Lymphatic vessel infiltration			*P *<* *0.0001			*P *<* *0.0001
Present	261 (36.7)	143 (61.1)		203 (32.3)	201 (62.4)	
Absent	450 (63.3)	91 (38.9)		420 (67.4)	121 (37.6)	
Venous vessel infiltration			*P *<* *0.0001			*P *<* *0.0001
Present	270 (38.0)	167 (71.4)		207 (33.2)	230 (71.4)	
Absent	441 (62.0)	67 (28.6)		416 (66.8)	92 (28.6)	
Vessel infiltration^2^			*P *<* *0.0001			*P *<* *0.0001
Present	339 (47.7)	192 (82.1)		266 (42.7)	265 (82.3)	
Absent	372 (52.3)	42 (17.9)		357 (57.3)	57 (17.7)	
Pathological TMN stage, *n* (%)			*P *<* *0.0001			*P *<* *0.0001
Stage I	448 (63.0)	71 (30.3)		420 (67.4)	99 (30.7)	
Stage II	124 (17.4)	79 (33.8)		102 (16.4)	101 (31.4)	
Stage III	99 (13.9)	61 (26.1)		69 (11.0)	91 (28.3)	
Stage IV	40 (5.6)	23 (9.8)		32 (5.1)	31 (9.6)	
Resection margin, *n* (%)			*P *=* *0.484			*P *=* *0.036
R0	679 (95.5)	221 (94.4)		600 (96.3)	300 (93.2)	
R1	32 (4.5)	13 (5.6)		23 (3.7)	22 (6.8)	

CAF, cancer-associated fibroblast; TEM1, tumor endothelial marker 1.

1 Nx: unknown for nodal status.

2 Vessel infiltration including venous vessel infiltration and/or lymphatic vessel infiltration.

### Kaplan–Meier curves show significant correlations between both CAF-TEM1-positivity and CAF-TEM1-intensity, and all-OS, cancer-OS, and RFS

**Figure 4:**
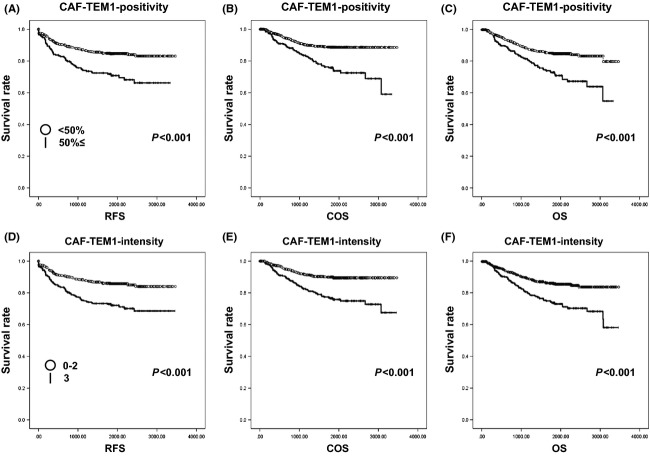
RFS curves, COS curves, and OS curves according to the grading of CAF-TEM1-positivity and CAF-TEM1-intensity. (A) RFS curves according to the grading of CAF-TEM1-positivity. (B) COS curves according to the grading of CAF-TEM1-positivity. (C) OS curves according to the grading of CAF-TEM1-positivity. (D) RFS curves according to the grading of CAF-TEM1-intensity. (E) COS curves according to the grading of CAF-TEM1-intensity. (F) OS curves according to the grading of CAF-TEM1-intensity. In (A–C), ○ and │ shows a CAF-TEM1-intensity of less than 50% (<50%) and a CAF-TEM1-positivity of more than 50% (50%≤) groups, respectively. While, in (D–F), ○ and │ shows the grade 0–2 and grade 3 groups for CAF-TEM1-intensity, respectively. RFS, recurrence-free survival; COS, cancer-related overall survival; OS, overall survival; CAF, cancer-associated fibroblast; TEM1, tumor endothelial marker 1.

The relationships between each of CAF-TEM1-positivity, CAF-TEM1-intensity, and vessel-TEM1-intesity, and all-OS, cancer-OS, and RFS were examined. Significant correlations between a CAF-TEM1-positivity of more than 50% and all-OS, cancer-OS, and RFS were observed when Kaplan–Meier curves were drawn (*P *<* *0.001) (Fig.4A–C). When the CAF-TEM1-intensity grading was divided into two groups (grade 0–2 and grade 3), significant correlations between CAF-TEM1-intensity and all-OS, cancer-OS, and RFS were observed when Kaplan–Meier curves were drawn (*P *<* *0.001) (Fig.4D–F). When vessel-TEM1-intensity was divided into any two groups, however, no significant correlations between vessel-TEM1-intensity and RFS, cancer-OS, or all-OS were observed when Kaplan–Meier curves were drawn (*P *=* *0.576, 0.123, and 0.515, respectively) (Fig. S1).

### Univariate analyses using the Cox proportional hazard model to identify TEM1-related factors influencing RFS, OS, and COS

Univariate analyses using the Cox hazard model to identify TEM1-related factors influencing the RFS, OS, and COS exhibited a significant association with a poor prognosis for CAF-TEM1-positivity and CAF-TEM1-intensity, as shown by the Hazard Ratio values (Table S2). However, vessel-TEM1-intensity was not significantly associated with a poor prognosis as shown in Table S2.

### Multivariate analyses using the Cox proportional hazard model to identify clinicopathological factors influencing RFS, OS, and COS

Multivariate analyses using the Cox hazard model to identify clinicopathological factors influencing the RFS, OS, and COS (Table S3) exhibited a significant association with a poor prognosis, RFS for CAF-TEM1-intensity, as had been shown by the Hazard Ratio values. However, venous vessel infiltration, pathological depth of penetration, and pStage were significantly associated with a poor prognosis, as previously known (Table S3). In addition, adjuvant chemotherapy was also significantly associated with a poor prognosis such as RFS and COS (Table S3) because these patients who received adjuvant chemotherapy were the patients with advanced gastric cancer associated with poor prognosis.

## Discussion

Cancer stroma is known to have diverse biological effects on cancer cells. Stromal cells, including fibroblasts, endothelial cells, and immune cells, have the potential to affect cancer cell progression; therefore, the molecules that stromal cells in cancer tissues, known as “CAFs,” specifically express could be suitable for molecular target therapy. In the current study, TEM1 was specifically expressed in the CAFs of gastric cancer tissue, and Kaplan–Meier curves exhibited a prognostic significance of CAF-TEM1-positivity and CAF-TEM1-intensity, with impacts on all-OS, cancer-OS, and RFS (*P *<* *0.001) (Fig.4). The possibility that TEM1 might be useful as a molecular target for the treatment of gastric cancer has not been previously reported. However, some reports have shown that TEM1 could be a target for the treatment of sarcoma. TEM1 expression was examined in 50 human tumor cell lines and 250 clinical specimens of human cancer, including 20 cancer subtypes 14. This previous report revealed that TEM1 was expressed in tumor cells, perivascular cells, and stromal cells in sarcoma 14. Moreover, the expression of TEM1 was examined in 11 types of carcinoma and the origin of the TEM1 expression was investigated 14. The results showed that TEM1 expression originated from the perivascular cells and stromal cells, and not from the carcinoma cells. In gastric cancer tissues, TEM1 expression was observed in none of the nine formalin-fixed, paraffin-embedded (FFPE) tissues when examined using immunohistochemical staining; however, TEM1 expression was detected in all seven frozen tissues using immunohistochemical staining, suggesting that TEM1 expression might be detected in the stromal cells of gastric cancer tissue samples in cases where the antibody was optimized for immunohistochemical staining using FFPE tissues 14. The antibody used in their report was not the same as that used in the current experiment; however, we validated the use of the anti-TEM1 antibody for immunohistochemical analysis using FFPE tissues through several experiments shown in Figures3. All the results showed that TEM1 was expressed in the stromal cells of gastric cancer tissues and was not detected in carcinoma cells in the other types of carcinoma, which was consistent with our other results. Another report using the Bioexpress database revealed one of two putative cancer stroma markers to be TEM1 15. These findings suggest that TEM1 expression can be detected not only in tumor cells originating from mesenchymal cells, but also in the stromal cells of cancer tissue.

The previous report showed that TEM1 expression was observed in sarcoma side population cells with stem cell-like properties 16, suggesting that TEM1 was a promising therapeutic target for sarcoma. In the current study, the chi-square tests showed that CAF-TEM1-positivity and CAF-TEM1-intensity were significantly correlated with several clinicopathological factors (Table2). Moreover, both a higher positive rate and a stronger intensity of TEM1 expression in CAFs were associated with poorer RFS, COS, and OS (Fig.4) and univariate analyses showed that CAF-TEM1-positivity and CAF-TEM1-intensity were significantly associated with RFS, OS, and COS, as had shown by the HR values (Table S2). These results suggest that TEM1 might also be a promising therapeutic target for carcinoma by targeting CAFs, rather than tumor cells, however, these TEM1 expression statuses in CAFs were not independent risk factors for RFS, OS, or COS (Table S3). Therefore, to clarify the mechanism by which the expression of TEM1 in CAFs affects the factors described above, further analyses will be needed using an innovative experimental animal model to study TEM1 biology arising from CAFs and to evaluate anti-TEM1 therapies.

TEM1 has so far been thought of as a promising molecular target for sarcoma; however, the present study showed the possibility that TEM1 inhibition might represent a new therapeutic strategy through the targeting of TEM1 expression in the CAFs of gastric carcinoma. This report is the first to suggest that anti-TEM1 therapy could benefit a wide range of cancer types including sarcoma and carcinoma.

## Supporting Information

**Figure S1.** Recurrence-free survival (RFS) curves, cancer-related overall survival (COS) curves, and overall survival(OS) curves according to the grading of vessel-TEM1-intensity.

**Table S1.** Relationship between vessel-TEM1-intensity and clinicopathological factors in 945 patients with gastric cancer.

**Table S2.** Results of univariate analyses using the Cox proportional hazard model to identify TEM1-related factors influencing RFS, OS, and COS in 945 patients with gastric cancer.

**Table S3.** Results of multivariate analyses using the Cox proportional hazard model to identify clinicopathological factors influencing RFS, OS, and COS in 945 patients with gastric cancer.
