# Supplementary figures and images for: TEM1 expression in cancer-associated fibroblasts is correlated with a poor prognosis in patients with gastric cancer
Source: Cancer Med. 2015 Sep 4;4(11):1667–78. doi: 10.1002/cam4.515 (PMC4673993; doi:10.1002/cam4.515)

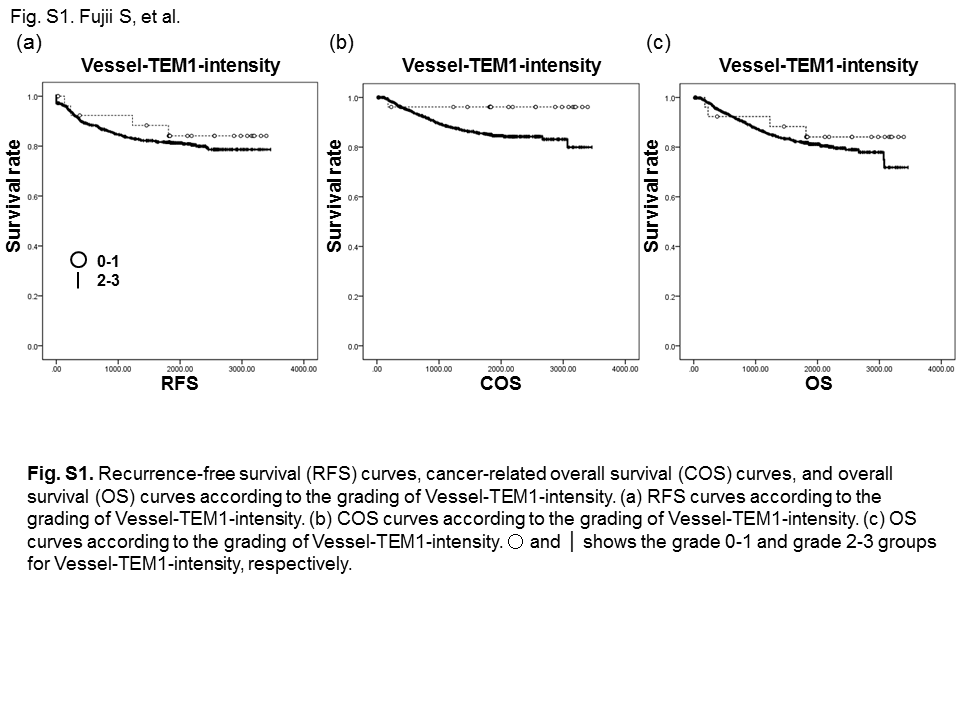

Supplement: Supplementary file 1 [file cam40004-1667-sd1.tif]
